# The prevalence of olfactory- versus visual-signal encounter by searching bumblebees

**DOI:** 10.1038/s41598-018-32897-y

**Published:** 2018-10-01

**Authors:** Jordanna D. H. Sprayberry

**Affiliations:** Muhlenberg College, 2400 W Chew St, Allentown, PA 18104 USA

## Abstract

While the phrase ‘foraging bumblebee’ brings to mind a bumbling bee flying flower to flower in a sunny meadow, foraging is a complicated series of behaviors such as: locating a floral patch; selecting a flower-type; learning handling skills for pollen and nectar extraction; determining when to move-on from a patch; learning within-patch paths (traplining); and learning efficient hive-to-patch routes (spatial navigation). Thus the term ‘forager’ encompasses multiple distinct behaviors that rely on different sensory modalities. Despite a robust literature on bumblebee foraging behavior, few studies are directly relevant to sensory-guided search; i.e. how workers locate novel patches. The first step in answering this question is to determine what sensory information is available to searching bumblebees. This manuscript presents a computational model that elucidates the relative frequency of visual and olfactory cues that are available to workers searching for floral resources under a range of ecologically relevant scenarios. Model results indicate that odor is the most common sensory cue encountered during search flights. When the likelihood of odor-plume contact is higher, odor-encounter is ubiquitous. While integrative (visual + olfactory) cues are common when foragers are searching for larger flowers (e.g. *Echinacea*), they become rare when foragers are searching for small flowers (e.g. *Penstemon*). Visual cues are only encountered in isolation when foragers are seeking large flowers with a low odor-plume contact probability. These results indicate that despite the multisensory nature of floral signals, different modalities may be encountered in isolation during search-behavior, as opposed to the reliably multimodal signals encountered during patch-exploitation or nectar/ pollen acquisition.

## Introduction

### Bumblebee populations are sensitive to decreases in foraging efficiency

Bumblebees are critical pollinators in both agricultural and native ecosystems^1–3^. Unfortunately these keystone species have experienced alarming declines alongside the highly publicized drops in honeybee numbers^4–7^. Critical work exposing the negative effects of neonicitinoid pesticides on bumblebee fitness indicates that pesticide exposure lowers rates of reproduction due, at least in part, to a drop in foraging efficacy of both workers and the colony as a whole^8,9^. This provides a critical link showing that the modification of worker behavior scales up to impact colony level fitness – a result that is consistent with seminal work showing that a colony’s ability to produce reproductive individuals is directly correlated with their size^10^. Better foragers provide more resources to rear young at the hive, which can increase the size of a colony during a foraging season. Given the current environmental pressures on bumblebees, developing a deeper understanding of their foraging behavior is relevant to conservation efforts.

### How do foragers search for flowers?

While the term “forager” can be defined as an animal locating and consuming food resources, it is a complicated series of behaviors. In bumblebees this includes: locating a floral patch^11,12^; selecting a flower-type^13,14^; learning handling skills for pollen and nectar extraction^15^; determining when to move-on from a patch^16,17^; learning within-patch paths (traplining)^12,18^; and learning efficient hive-to-patch routes (spatial navigation)^11,12,19^. Thus the term ‘forager’ encompasses multiple distinct behaviors that rely on different sensory modalities^20–22^. A critical component of foraging theory is the search phase^23^; which would be floral patch location in the case of pollinators. This phase is comprised of: (1) movement through the environment; and (2) recognition of resources, which should terminate the search. There is a wealth of literature analyzing forager search paths, from bumblebees to albatrosses^24–27^. While there is some controversy over the precise algorithms that accurately describe these search paths^28–33^, there is consensus that search paths can be reasonably represented with stochastic models of forward-biased motion (i.e. while turning events happen, complete direction reversal will be rare). Once a searching forager recognizes a resource, their behavior should transition from random-search to approach and feeding. In bumble bees the ability to recognize floral resources will be dependent upon perception of floral signals. Flowers provide complex sensory displays, including color, shape, nectar guides, odor and morphology^34–37^. In the case of pollinators searching for novel patches, only those sensory cues capable of operating at a distance will factor into recognition and subsequent sensory-guided navigation. Morphological cues are only relevant upon physical contact with flower and are thus not useful for search. Complex patterns on flowers, such as nectar guides or visible stamens, are only resolvable at close distances (4–45 cm)^38^. Thus shape, color and odor are the sensory signals most likely to be available for patch recognition.

### Odor pollution impacts forager behavior, but the effects on foraging efficiency are unclear

Several studies over the past decade have indicated that anthropogenic odor pollution is both modifying floral odor plumes^39^ and subsequent behavioral responses of bees^40,41^. While this work is interesting from a neuroethological standpoint, it is currently unclear how drastically natural foraging populations are impacted by odor pollution. Understanding the potential impact of odor-pollution first requires an understanding of odor’s role in foraging.

There is a substantial body of work indicating that olfaction is important in patch exploitation; however, the precise role that odor plays is not completely understood. PER studies indicate that bumblebees are capable of associative odor learning^42–44^, generating the logical hypothesis that floral odor could be used to identify rewarding flowers. Multimodal studies investigating both vision and olfaction indicate that stimulation of odor pathways improves foraging accuracy, regardless of whether or not floral signals have differentiating odor stimuli^45^. Field experimentation on floral morphs showed that bumblebees prioritized visitation of a learned visual (color) signal over the learned odor^46^. These findings might imply that any odor is effective, and that precise odor identity might be irrelevant. However, work by Leonard *et al*. showed that when flowers differ in both visual and olfactory modalities, foraging accuracy was higher^35^ – pushing back against the idea that odor identity is unimportant. Social odor cues – tarsal scent deposits on flowers, reduce bumblebee visitation rates. This is an example of a ‘contaminating odor’ that increases energy gain by reducing visitation to recently emptied flowers. It is likely that scent marks are perceptually distinct from the floral odor, rather than modifying the floral blend-structure such that it becomes unrecognizable to the bumblebee, as behavioral data have been relatively consistent across multiple flower species^47^ and with unscented artificial flowers^48^. Therefore, it appears that the precise odor identification of tarsal scent-marks is quite important to foraging behavior. Given the contradictory nature of current data on odor usage, it is difficult to predict the effects of pollution on foraging efficiency during patch exploitation.

There is a paucity of work looking at the impact of odor on navigation to food resources in bumblebees. Several lab-based studies indicate that odor alone is sufficient to facilitate navigation^40,49^. However, the relative roles of odor and vision (which could have implications for how drastic the effects of odor pollution might be) have never been investigated at a spatial scale that would shed light on the role of odor in patch location. For example, lab studies are typically in arenas that are less than 3.6 m in their largest dimension^13,35,40,48,50–59^. However, the foraging range of a bumblebee can reach up to 1.75 km from their nest^60^– a distance that is orders of magnitude larger than typical sensory-behavior studies, even those that are based in the field^46^. An understanding of odor-pollution’s impacts requires a better understanding of the relative roles that vision and olfaction play in navigation to floral resources. If a searching-forager is consistently encountering an odor signal before a visual signal, it stands to reason that odor-guided navigation will bring that animal within visual range of a flower. Given that odor plumes are theoretically available at a much greater distance from a flower^39,61^ than visual cues^38,62^ this is a logical assumption. However, odor plume contact is stochastic, and some empirical measurements of odor-plumes indicate much shorter distances travelled^63^. This manuscript presents a computational model that moves beyond assumptions and asks *– given the probabilistic nature of odor plume contact- what is the likelihood of a bumblebee encountering resolvable visual versus olfactory cues?*

## Methods

**Figure 1:**
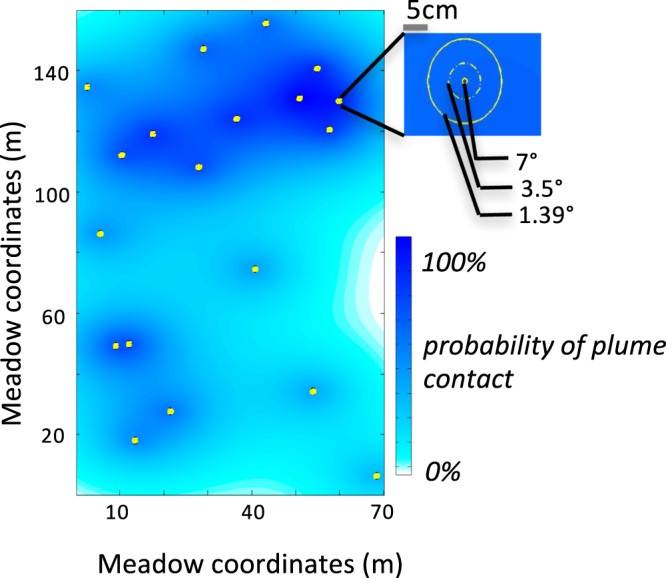
Sensory construction of the artificial meadow that ‘bumblebees’ searched within. The digitized meadow from a scenario utilizing single-flower *Echinacea* plants, a plant density of 1/600 m^2^, and a high odor probability. The probability of odor contact is represented by the blue contours. The visual resolvability is represented by the yellow circles; the inset labels the relationship between angular resolution of the searching bumblebee and the distance at which a flower becomes visible. The finest resolution (1.39 degrees) results in the greatest visual detection distance.

In order to determine which sensory cues are available to searching foragers this model creates a random search path for a bumblebee through a simulated meadow and at each step assesses whether or not the bumblebee has encountered a resolvable visual or olfactory cue from flowers populating the meadow. Meadow dimensions (70 m × 160 m) were based upon Google-satellite images of a clearing at Conrad W. Raker Sanctuary, a biological field station owned by Muhlenberg College (Fig. 1).

### Bumblebee Movement

This model generated a search path for bumblebees in order to walk them through a digital meadow until they encountered a salient and resolvable floral sensory cue. These computational paths utilized a correlated random walk model (CRW) to generate motion in the latitude-longitude plane:

$$\begin{array}{rcl}\alpha (t+T) & = & \alpha (t)+\beta (t)\,\\ x(t+T) & = & x(t)+l\cdot \,\cos \,(\alpha (t))\\ y(t+T) & = & x(t)+l\cdot \,\sin \,(\alpha (t))\end{array}$$

where: **α** is the current heading angle. **β** is the turning angle, where the probability of turning is based upon digitization of Heinrich’s canonical observations of bumblebee foraging behavior^64^ (Table 1). **T** is the time step – an iterative and scale-less variable whose actual value is represented by step length. **x(t** + **T)** gives the longitudinal position of the bumblebee in the next time step. **y(t** + **T)** gives the latitudinal position of the bumblebee in the next time step. **l(t)** is a step length of 0.3 m; determined by the product of bumblebee flight speed (3 m/s^65^) and the interspike interval of motor neurons (approx. 0.1 s^66^). This represents a reasonable estimate of how rapidly the flight system could change course.

**Table 1:** Turning probability digitized from Fig. 3 in Heinrich^64^.

Turn angle (degrees)	Probability (0–1)
−135	0.025
−90	0.065
−45	0.19
0	0.44
45	0.19
90	0.065
135	0.025

This method of search-path computation deviates slightly from the more commonly-referenced Levy walk^19,24,26–28,67,68^ in that the step length is constant, rather than pulled from a power-law distribution^29^. Maintaining this constant step length allows the model to be tightly parameterized to known flight-speed measurements (as in Becher *et al*.^69^) rather than incorporate occasional large step lengths that imply biologically implausible flight speeds. Work by James *et al*. indicates that efficiency in resource location by searching foragers has little to do with the search algorithm and is predominantly driven by the density of food resources^70^; if so the use of a CRW in this model should not corrupt the results. In addition, the basis for Levy-flights/walks being a behaviorally accurate method of modelling forager-search behavior has recently been called into question^31,33^. However, given the prevalence of Levy flights in foraging literature, it is worthwhile to confirm that using this method would not significantly modify conclusions about floral sensory encounter. Thus, a subset of model-conditions were run with variable step lengths drawn from a Levy Distribution^67^:

$$P(l)={(\frac{l}{{l}_{0}})}^{-\mu }$$

where: *P*(*l*) is the probability of a particular step length. *l* is the corresponding step length. *l*_0_ is the minimum step length, set to 0.3 m (see justification above). *μ* is an exponential constant such that if it is between 1 and 3 the distribution meets the requirements for a Levy flight/walk. In this case it is set to 2, which produces an optimal search strategy^71^.

Individual model runs started with bumblebees entering the meadow at a randomized edge location – mimicking arriving at the meadow from adjacent wooded territory. In all cases individual model runs continued until the bumblebee encountered a resolvable sensory signal (see *Sensory Performance of Bumblebees*) or completed 5000 steps – the equivalent of 1.5 km in the CRW, a value selected because it falls in the upper range of measured foraging distances^60^. Given the variable step lengths in the Levy-flight condition, each model run has a unique potential maximum distance travelled; the mean for 1000 runs is 3 km.

Because this model ends its runs upon floral-*signal* encounter, it is investigating what sensory information is available to searching bees and does not explicitly simulate floral approach. However, given that the visual acuity measurements are derived from behavioral rather than physiological experiments^38,62^ bumblebees should be able to visually navigate to a “found” flower. Likewise, there is a body of work indicating that bumblebees are capable of using odor cues at a distance to locate food resources^40,49^. It is therefore plausible that searching bumblebees would be capable of acting upon salient and resolvable sensory stimuli; i.e. workers would be able to pick up where the model leaves off. This model does not model floral approach because it is outside the scope of our current question.

### Floral Parameters

**Figure 2:**
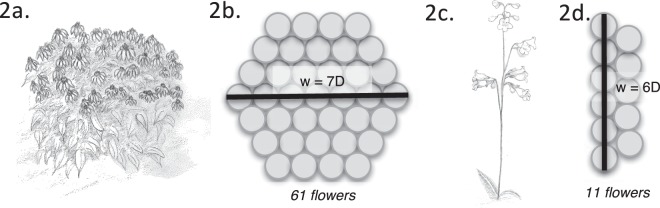
The relationship between flower number and the size of visual stimulus (w). The morphology of *Echinacea* plants (**a**) lends itself to polygon packing of blooms (**b**) while the upright habit of *Penstemon* (**c**) makes a double row arrangement (**d**) a more logical choice for that species.

Given that bumblebees are likely to encounter environmental variation in the field, the model varied plant density, plant size, and inflorescence size. Floral parameters were based upon published data from *Echinacea* and *Penstemon spp*, two common native genuses with wide ranging distributions that are readily pollinated by bumblebees^72,73^. These species provide an ecologically relevant range of bloom sizes, ranging from 0.7 cm diameter (*Penstemon*^74^) to 7.6 cm (*Echinacea*). Previous work on *Echinacea spp* indicates a wide range of naturally occurring densities (0.001 to 3 plants/m^2^, estimated from nearest neighbor data in Wagenius and Lyon^72^). For the purpose of this study, which is interested in navigation to novel patches, I tested a realistic range of low densities (i.e. situations where the next nearest patch was not likely to be within visual range of the first): 1 plant/6 m^2^ (0.17), 1 plant/60 m^2^ (0.017), and 1 plant/ 600 m^2^ (0.0017). Field data on *Penstemon* indicated higher density tendencies, with a range of 0.16–1.64 plants/m^2^. Thus the total set of tested densities was 0.0017, 0.017, 0.17, 0.89, 1.64. Floral patches were then randomly distributed throughout the meadow based upon the overall density (the number of plants per square meter). A brief survey of *Echinacea purpurea* plants revealed a high variability in number of blooms per plant (3–62). Therefore, I tested five different display sizes based on number of observed flowers: 1, 7, 19, 37, and 61. To determine the diameter of these displays flowers were polygon packed, resulting in diameters of 0.076, 0.228, 0.38, 0.532, and 0.684 meters respectively (Fig. 2a,b). *Penstemon* plants have a different growth habit than *Echinacea*, presenting flowers vertically on spikes; therefore, I used a two line packing of multiple blooms to estimate display size from the largest dimension (Fig. 2c,d). Recent work has shown the mean daily number of flowers for *Penstemon digitalis* to be 5^75^– the tested number of flowers were 3, 5, 7, 9, and 11 to encompass a range around this mean; resulting in display sizes of 0.014, 0.021, 0.028, 0.035, and 0.042 meters respectively.

### Sensory Performance of Bumblebees

Bumblebee size has a marked effect on visual acuity^62^. Given the large variability in worker size that is likely to occur in natural populations, the model was tested with three different visual acuity values (1.39, 3.5, and 7 degrees) representing the range of values in the literature for two bumblebee species (*Bombus terrestris*^62^ and *Bombus impatiens*^38^). Flowers were considered to be resolvable if their angular size from the bumblebee’s current position was equal to or greater than the visual acuity value. Angles were calculated as:

$$\theta =ta{n}^{-1}(\frac{w}{D})$$

where: *θ* is the angle subtended by the floral display. w is the width of the floral display. D is the bumblebee’s distance to the floral display calculated via the Pythagorean theorem.

While the CRW generates planar motion, bumblebees clearly forage in three dimensional environments. Therefore, distance calculations assumed bees were flying 1 m above vegetation.

**Figure 3:**
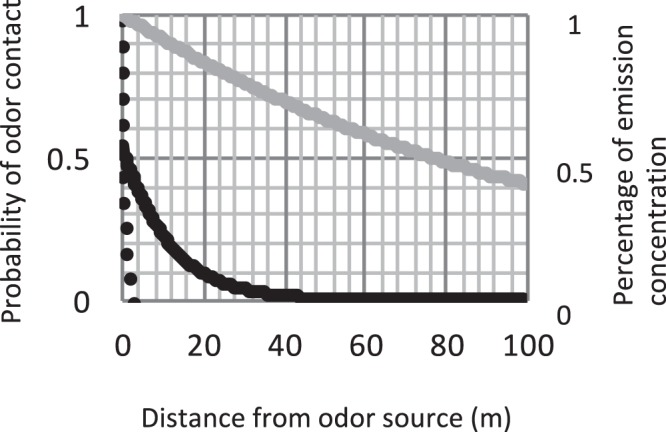
The black lines represent the relationship between distance from flower (odor source) and the likelihood of plume contact, with the solid line representing a ‘high’ probability derived from Murlis *et al*.^64^ and the dashed line representing a ‘low’ probability derived from Riffell *et al*.^67^. The grey line is an estimated fit derived from McFrederick *et al*.’s^48^ calculations on distance travelled by floral odorants, represented by percent of emission concentration. By these calculations common floral odorants do not drop below 80% of original concentration until 25 m from source, indicating the odor probabilities used in this model are quite conservative. Additionally, comparing the concentration decay with the model’s odor probabilities indicates that when bumblebees have a 10% probability of plume contact, that plume is still at >80% original concentration, thus that plume is likely physiologically salient.

While variation in body size of workers also impacts olfactory performance^49^, the resolution of olfactory stimuli is based upon the likelihood of encountering an intact (i.e. not well mixed and therefore diluted) and resolvable plume-filament. Probability of plume contact is derived from studies investigating odor plume availability in field conditions; this model is making the assumption that an odor filament strong enough to be measured by laboratory equipment would be strong enough to stimulate a response. Given work by Murlis *et al*.^61^ – where they found a 1:1 relationship between antennal response in *Manduca sexta* and presence of a measured plume – this is a physiologically reasonable assumption. As existing field measurements show variability across environments, I tested two different olfactory probability functions (Fig. 3). The ‘high’ probability function was based upon field measurements by Murlis *et al*. taken in mid-July in an open field near Amhurst, MA^61^. This study provided field measurements of contact probability up to a distance of 20 m. However, odor plumes tend to be highly mixed and thus undetectable by a distance of 100 M^76^. Thus I bookended the Murlis data with two constraints: a value of 1 at 0 meters, representing the maximum probability of plume contact; and a value of 0.0001 at 100 meters – given that the model was repeated 1000 trials per condition this value functionally represents zero. These data were then fit with an exponential in excel (y = 0.54e^−0.086×^, R^2^ = 0.99, Fig. 3). The ‘low’ probability was taken directly from the exponential fit from Riffell *et al*.’s work measuring plume structure in a high alpine desert^63^ (Fig. 3). Odor probabilities operated in a radially symmetric fashion around floral displays (Fig. 1). Both of these probabilities are based upon data from studies on the hawkmoth *Manduca sexta*, a model organism in the study of odor-guided flight^77^ and olfactory processing^78–80^. Hawkmoths have both a larger body size and antennal length than *Bombus* species^81,82^. Given that body size in bumblebees correlates with greater olfactory sensitivity^49^, there is the possibility that odor-encounter probabilities for *Manduca* over-estimate bumblebee olfactory capabilities. Interestingly, a comparison of the odor-behavior literature shows that bumblebee experiments are typically run at much lower odor concentrations (1:1000) than *Manduca* experiments (neat extracts)^40,49,80,83^. In addition, bumblebee electroantennogram experiments (EAGs) require significantly higher odor concentrations (1:10–1:100) than bumblebee behavior experiments^49^ (*Sprayberry unpub data*), likely due to the noisy nature of electrophysiology recordings requiring a stronger stimulus to create a favorable signal: noise ratio. This is relevant because the “high” probability odor encounter plume is derived from EAG recordings, and thus likely underestimates the actual sensing ability of insect antennae. Therefore, while *in-vivo* hawkmoths may have higher odor sensitivity than bumblebees, the anthropogenically-derived odor probabilities are likely applicable to both insect groups. In addition, even the high-probability fit is conservative when compared to calculations of distance travelled by floral odorants, which indicate a less than 50% loss of volatiles at distances of 100 m^39^ (Fig. 3). McFrederick *et al*.’s computational analysis does not consider plume structure – those remaining molecules may be well mixed and thus at physiologically irrelevant concentrations; however, it does imply that this model is unlikely to overestimate olfactory contact.

## Results and Discussion

This model explored the sensory signals available to bumblebee foragers searching for novel resources by calculating the relative probability of workers encountering the visual and/or olfactory signal from a floral resource while searching in a relatively low-resource environment. The parameters varied in this model were: plant density, number of blooms (and thus the strength of sensory signals from an individual plant), the probability of odor plume encounter, and the visual acuity of the searching “bumblebee”.

### Olfaction is the dominant sensory modality available to searching bees

**Figure 4:**
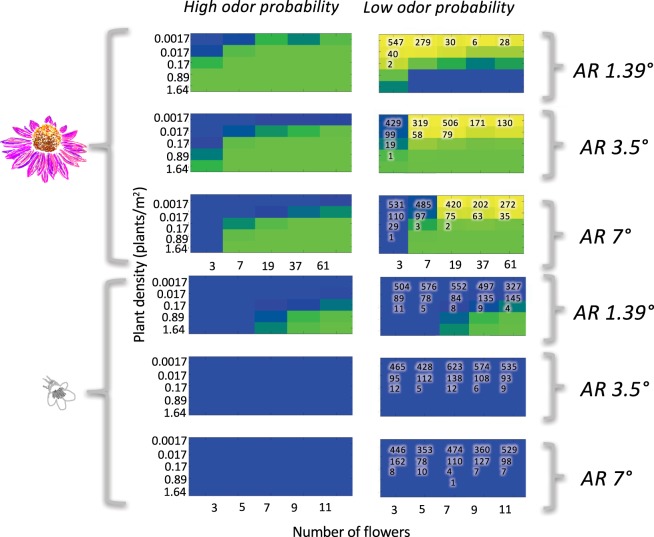
Heat maps indicating the relative likelihood of encountering a resolvable olfactory (blue), visual (yellow), or integrated olfactory and visual (green) sensory signal. These likelihoods were calculated for:1. multiple plant sizes, indicated by a variable number of flowers on the x axes; 2. multiple plant densities, indicated on the y axes; 3. two different plant species, *Echinacea* (top diagram) and *Penstemon* (bottom diagram); 4. two different odor probabilities, with high encounter probability represented in the left row and low on the right; and 5. three different visual acuities, labelled with their angular resolution on the right hand side of the figure. Each model scenario was run 1000 times. The number of failures – runs where a bee searched for 1.5 km without encountering a sensory signal- are indicated on the plots themselves. The absence of a number means that all 1000 runs resulted in a successful sensory encounter.

**Figure 5:**
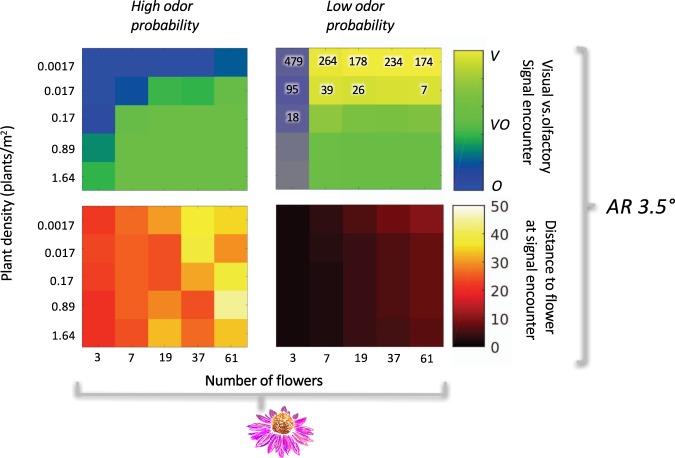
Results of model runs using a Levy-walk distribution of step lengths for bumblebees with a visual resolution of 3.5*°* searching for *Echinacea*. Despite the difference in search-path calculation methods, the results are nearly identical to those depicted in Figs 4 and 6. Levy-walk searches do lead to a slight reduction in failure rates for low-odor probability scenarios.

Looking holistically at all tested scenarios we see that odor dominated as the available sensory modality; with odor alone representing floral sensory encounter in 179/350 scenarios, an integrated odor-visual signal available in 136/350 scenarios, and vision alone as the dominate modality in only 35/350 (Figs 4 and 5). Odor information is therefore available for decision making in 90% of successful floral encounters, while visual information is only present in 49%. While there is substantial work indicating that vision is vitally important for patch exploitation behaviors^35,46,84^, it is likely that odor is crucial in patch location behavior.

### Odor landscapes are changing, which could have a considerable impact on bumblebee foraging behavior

**Figure 6:**
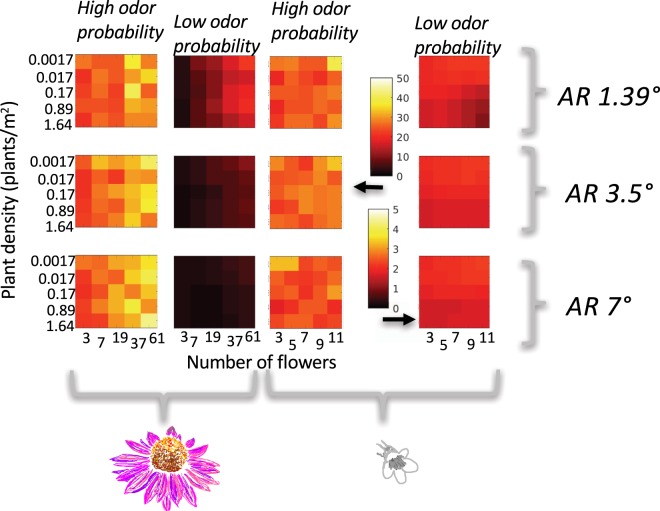
Heat maps indicating average distance at which a resolvable sensory signal was encountered in *successful* model runs. These likelihoods were calculated for: 1. multiple plant sizes, indicated by a variable number of flowers on the x axes; 2. multiple plant densities, indicated on the y axes; 3. two different plant species, *Echinacea* (two left columns) and *Penstemon* (two right columns); 4. two different odor probabilities (labelled by column); and 5. three different visual acuities, labelled with their angular resolution on the right hand side of the figure.

Model runs with a higher probability of odor contact demonstrated a larger discovery distance, with bumblebees contacting a resolvable sensory signal in the range of 25–40 meters, as opposed to 2–20 meters (Fig. 6). Additionally, decreased probability of odor-contact drastically increased the likelihood that forager searches would end in failure (Figs 4 and 5). Failure rates overall were higher for the smaller bloom size (*Penstemon*), as the larger plant and flower size of *Echinacea* afforded a better ability to transition to visual navigation when odor was unavailable. These computational results are commensurate with laboratory investigations on visual search time in bumblebees, where bees who have been restricted to solely visual information have higher search times to locate smaller flowers^85^. The low-odor probability tested in this model decays rapidly, transitioning to zero before 10 meters from the point source (Fig. 3)^63^. This empirical measurement may be underestimating plume strength due to environmental conditions: previous work has shown that odor plumes can rise in altitude^86^ and the Riffell *et al*. measurements were taken at a consistent elevation from the ground. However; the results from this odor fit are relevant to consider in light of work examining the impacts of anthropogenic pollution on floral odor-plumes. Seminal work by McFrederick *et al*. indicates that environmental pollutants can interact with floral odorants, reducing their distance travelled by an order of magnitude drop: odorants that historically could travel 1000 m before dropping to 80% of their original concentration would only make it 100 m in worst case scenarios. While McFrederick’s study was computational, subsequent experimental studies have been equally concerning. Girling *et al*. found that diesel exhaust degrades select floral odorants, modifying odor blend structure^87^. Likewise Farre-Armengol *et al*. found that ozone decreases floral odorant concentrations^41^. Based on our model results it is reasonable to hypothesize that bumblebees will experience higher failure rates in locating flowers when searching in polluted environments, particularly if available floral resources are comprised of plants with smaller bloom size and lower bloom number. Indeed, failure to locate a floral signal only occurred in the low-probability odor scenario - when odor information is readily available searching events are universally successful.

### Bloom size, number of blooms per plant and plant density impact both available sensory modality and distance at which plants are found

Unsurprisingly, plant size and density impact the likelihood that bumblebees will encounter a resolvable sensory signal (Figs 4 and 5). Increased plant density reduced failure rate in low odor probability situations for both large (*Echinacea*) and small (*Penstemon*) flowers. However, when plants with small bloom sizes are in low density patches they were only reliably ‘found’ in model runs with a higher probability of odor plume contact. *Echinacea* simulations were moderately less susceptible to density effects as they can be seen from a greater distance, but higher flower number was still associated with an increased discovery distance. Interestingly, field data on *Penstemon* indicated that they were typically found at the higher densities this model tested – the lower densities tested here were included purely for comparative purposes. In light of anthropogenic modulation of odor environments, bumblebees may passively select for larger bloom size and higher plant density in polluted environments by virtue of not being able to locate smaller flowers, or those with larger nearest-neighbor distances.

### Effects of search-path type

The outcome of model runs using a power-law distribution for step lengths (Levy-walk) (Fig. 5) is nearly identical to the results from constant step lengths (Fig. 4). Odor information is ubiquitous in the high-odor probability scenarios, with visual information not being encountered in isolation until the low-odor probability scenarios. As in Figs 4 and 6, a shift to low-odor probability both decreases the distance at which flower-signals are encountered and increases failure rates in search flights. The predominant difference between the two search-path methods is a slight decrease in failure rates when using variable step lengths, a finding that is consistent with the fact that the latter method ran for approximately double the distance, creating a longer search path.

### Limitations and Future Directions

It is worth emphasizing that this experiment was done in silica. As such it is limited by the assumptions used to generate model results. These model results are strongly driven by visual and olfactory resolution: on the plant side from the strength of floral signal, and on the pollinator side from sensory sensitivity. While all of these variables were parameterized based on the ecology and physiology of the relevant plant-pollinator relationships, the absolute values returned by the model are less relevant than the trends. These trends raise interesting questions for future experimental work. For example, the indication that bumblebees with lower visual acuity first encounter smaller floral displays via odor plumes begets the question, will bumblebees searching for novel resources navigate with odor information alone? This phenomenon has previously been demonstrated on a small spatial scale^40,49^, but remains to be shown at field-realistic scales. The substantial number of model runs finishing with simultaneous odor and visual signal encounter raises the question, does odor information make a minimally resolvable visual cue more salient? Again, work on small spatial scales shows improved learning and recognition of food resources with multimodal sensory information^51,88^, but how this operates on large spatial scales is less clear. Finally, this computational model provides an alarming context for recent work on odor pollution in bee behavior. While that work has largely focused on laboratory investigations, decreasing plume distance is likely to have profound impacts on foraging efficiency in bumblebees and other odor-guided pollinators. These results, in combination with recent computational findings on air pollution decreasing distance travelled by floral scent^67^, strongly indicate that relevant field work to ground-truth theoretical concerns is necessary.

## Data Availability

Data generated by model runs are available upon request.
